# Faith without action? Christian ecological conviction and the civic participation gap in Australia

**DOI:** 10.1007/s13280-026-02388-w

**Published:** 2026-04-21

**Authors:** Timothy J. Healy, Johannes M. Luetz, Martin J. Hodson

**Affiliations:** 1 School of Ministry and Theology, Alphacrucis University College, Perth, WA 6103 Australia; 2 School of Social Sciences, University of New South Wales, Sydney, NSW 2052 Australia; 3 Graduate Research School, Alphacrucis University College, Brisbane, QLD 4102 Australia; 4 School of Law and Society, University of the Sunshine Coast, Maroochydore, QLD 4556 Australia; 5 Department of Biological and Molecular Sciences, Faculty of Health and Life Sciences, Oxford Brookes University, Oxford, UK

**Keywords:** Belief–behaviour gap, Christianity and environment, Civic engagement, Faith and ecology, Pro-environmental action, Sustainability transitions

## Abstract

Three-quarters of the world’s population identify with a religion, suggesting that faith communities constitute a vast (yet under-mobilised) civic infrastructure for sustainability transitions. Christianity remains the world’s largest religious tradition, making it consequential for analysing conviction–action dynamics. Using Australia as a case study, this research investigates why widely professed moral concern for the environment does not translate more readily into collective environmental action. Using Australia as a case study, our national sample survey (*n* = 1295), weighted to reflect the Australian population aged 18+, indicates a belief-action gap that is systematic rather than incidental, underpinned by barriers across micro-meso-macro levels. Our analysis identifies actionable strategies to help faith communities translate ecological conviction into effective civic engagement with policy-relevant sustainability outcomes. By clarifying pathways through which faith commitments can be mobilised into civic action, the article offers insights transferable beyond Australia and churches to other national and faith contexts, with implications for advancing SDGs 3, 6, 11, 12, 13, 16, and 17.

**Supplementary Information:**

The online version contains supplementary material available at https://doi.org/10.1007/s13280-026-02388-w.

## Introduction

The accelerating urgency of climate change has intensified attention on the social drivers of pro-environmental behaviour and collective action. This question is not marginal. Today, roughly three-quarters of the world’s population (about 76%) identify with a religion, including an estimated 2.3 billion Christians and 2.0 billion Muslims. Some broader demographic estimates place this figure higher, at up to 85% of the global population, underscoring faith as a pervasive social force with direct relevance for sustainability transitions (Schliesser 2024). Christians and Muslims together account for more than half of humanity, and Christianity alone remains the world’s largest religious tradition (Hackett et al. 2025).

From a sustainability science perspective, the question of how religious conviction translates (or fails to translate) into pro-environmental and climate-positive behaviour is therefore not merely of theological interest. It represents a significant opportunity space for accelerating societal responses to climate change, particularly where faith communities function as durable institutions of norm formation, trust, and collective mobilisation (Clarke 2016). Recent research at the religion–sustainability nexus shows that faith traditions often shape knowledge as well as ethics, influencing how environmental change is interpreted, which claims are treated as credible, and how moral responsibility is construed (Luetz 2025, 2026). In practical terms, faith-based mobilisation has the potential to advance the SDGs by contributing to health and well-being co-benefits (SDG 3), water stewardship and sanitation-related sustainability outcomes (SDG 6), and the protection of culturally significant places and communities (SDG 11), while also supporting responsible consumption and more sustainable everyday practices (SDG 12), strengthening climate mitigation and adaptation action (SDG 13), supporting legitimate and trusted civic institutions that enable collective action (SDG 16), and scaling cross-sector partnerships between religious organisations, government, and civil society (SDG 17).

In high-income contexts such as Australia, private, low-cost household actions (such as recycling or switching off lights) are now widespread, yet civic and collective engagements (for example, contacting elected officials, donating to environmental organisations, or participating in local conservation activities) remain comparatively rare (Whitmarsh et al. 2021). This divergence points to a persistent social–environmental challenge: the translation of environmental concern and moral commitment into forms of participation that can influence institutions, policies, and collective sustainability outcomes.

Religious communities, and Christianity in particular, in the present study, are often assumed to provide moral motivation for ecological responsibility (Clarke 2016; Edvardsson Björnberg and Karlsson 2022). Yet emerging evidence suggests that the relationship between faith and action is more complex. While some Christians articulate stewardship, neighbour-love, and climate justice as core faith commitments, these convictions are unevenly enacted and are not reliably expressed as consistent pro-environmental behaviour across the wider Christian population (Malcolm and Scott 2025).

Here, we argue that Christian ecological conviction does not reliably translate into pro-environmental practice. Drawing on a national sample survey (*n* = 1295), weighted to reflect the Australian population aged 18+, we show that while nearly three in ten Australians—and a substantially higher share of Christians who attend church regularly—agree that Christian faith encourages ecological responsibility, Christians overall are no more likely than non-Christians to report either routine pro-environmental practices or civic action on environmental issues. Civic engagement is uncommon overall, and on some measures, Christians are even less likely than other Australians to participate. We argue that this belief–action gap is systemic rather than accidental: it reflects cognitive barriers (including perceived inefficacy and familiar “dragons of inaction”), weak or ambiguous congregational norms for public engagement, and a lack of institutional scaffolding that could make civic participation easy, legitimate, and routine.

Australia offers an analytically useful case for examining faith–action gaps in environmental engagement because it sits within a cluster of Anglophone settler democracies with broadly comparable sociocultural identities and institutional configurations, including the USA (Vucetic 2011). As such, the mechanisms identified here are internationally transferable to similarly configured contexts—many of which have been historically consequential contributors to global warming and prominent arenas of climate politics and public discourse (Hickel 2020).

This article contributes to, and seeks to advance, interdisciplinary research on the conviction–action gap, religion and ecology, and sustainability transitions. Specifically, we shift attention from individual attitudes to the meso-level infrastructures of congregational life, and we propose a set of testable mobilisation levers through which faith communities could translate conviction into civic participation with meaningful sustainability outcomes.

We proceed as follows: Sect. “Theoretical framework and literature review—Faith and the attitude–behaviour gap: Insights from recent scholarship” reviews relevant literature on private environmental behaviour, civic engagement, and religion and ecology; Sect. “Materials and methods” describes the survey dataset, sampling approach, analytic categories, and weighting procedures; Sect. “Results—The faith–action disconnect in Australia” presents survey evidence on the faith–action gap among Australian Christians; and Sect. “Discussion—Explaining and addressing the disconnect: Pathways to civic action” develops an explanatory framework and outlines seven congregation-level mobilisation strategies with relevance beyond Australia and churches; and Sect. “Conclusion—The way ahead” discusses implications, limitations, and future directions for research and practice.

## THEORETICAL FRAMEWORK AND LITERATURE REVIEW

### Faith and the attitude–behaviour gap: Insights from recent scholarship

Despite increasing awareness of environmental issues and the growing prominence of sustainability discourse, a substantial disjunction persists between individuals’ values and beliefs and their actual ecological behaviours. This inconsistency is frequently described in the scholarly literature as the value–action gap or the belief–behaviour gap. The terms refer to the phenomenon in which individuals espouse strong environmental values or beliefs yet fail to engage in consistent pro-environmental behaviours. Understanding this paradox is crucial, as it facilitates the development of tailored policy interventions, corporate strategies, and community-based initiatives that can bridge the gap between expressed values and actual conduct. This section examines current academic perspectives on these gaps, their underlying causes, and their manifestations. The review synthesises findings from studies conducted within the last five to ten years to investigate this phenomenon across various domains.

Consumer studies repeatedly document intention–behaviour inconsistencies: many people report pro-environmental values but do not consistently act on them (Grimmer and Miles 2017). Global opinion tracking similarly shows that most consumers say sustainability matters, but comparatively few change purchasing or energy-use habits in practice (Ipsos 2021; Yang et al. 2026). Recent Ipsos data report that although around 80% of respondents express concern about climate change, only about a third believe their own actions can make a meaningful difference. This pattern suggests declining confidence in the efficacy of individual action and a corresponding expectation that governments and corporations should take primary responsibility (Ipsos 2023). This “diffusion of responsibility” is central to the value–action gap in environmental sustainability: when responsibility is experienced as external, shared, or primarily institutional, individuals feel both morally licensed and practically disempowered, and inaction becomes easier to justify (Gifford 2011).

The gaps are frequently elucidated in the literature through models derived from psychology and behavioural economics, with numerous studies probing the psychological mechanisms underpinning the discrepancy between values and actions (White et al. 2019; Whitmarsh et al. 2021). Gifford’s concept of “dragons of inaction” synthesises other psychological barriers such as limited cognition, perceived risks, conflicting objectives, and tokenism (Gifford 2011). More recent research underscores the significance of temporal psychology: individuals who perceive themselves as responsible for future generations demonstrate stronger, more consistent pro-environmental engagement (Syropoulos and Markowitz 2024).

Cost salience, convenience, and basic choice architecture can all suppress pro-environmental intentions. Lane et al. (2025) report that although Australian households endorse circular-economy ideas such as repair and reuse, they rarely act unless there is a clear financial benefit. Sector-specific work on green electricity also shows that uptake depends not only on environmental values but on perceived reliability, pricing, and trust—factors that can be shaped through policy and design to lower hassle costs and make the greener option feel like the obvious default (Yang et al. 2026). Large-scale international survey evidence (> 130 000 respondents across 180+ countries) similarly finds that low-cost behaviours (e.g. reducing food waste or meat consumption) are far more common than high-cost shifts (e.g. installing heat pumps or buying electric vehicles). People who have not adopted low-cost behaviours tend to cite lack of information, whereas those who have not adopted high-cost behaviours most often cite expense or incompatibility with household needs (Leiserowitz et al. 2024).

Behavioural reviews emphasise that durable pro-environmental change depends on aligning motivation with contextual changes that lower friction and make the sustainable option easy to choose (Whitmarsh et al. 2021). The SHIFT framework (Social influence, Habit formation, Individual self, Feelings/cognitions, Tangibility) operationalises this logic into practical levers for influencing behaviour. It treats value alignment as necessary but not sufficient, and instead locates behaviour at the intersection of belief, context, and capacity (White et al. 2019).

Cultural factors also play a role in shaping the value–action gap. Bai et al. (2025) provide cross-cultural evidence from China and the UK, indicating that societal expectations and collective cultural values markedly influence the manifestation of the value–action gap. In more collectivist settings, people are more likely to act when there is visible normative encouragement and a sense that “people like us do this”. Related longitudinal research finds that prosocial online communities—including collaborative gaming spaces—can catalyse environmental activism by building shared identity and perceived collective efficacy (Shliakhovchuk et al. 2025). Together, these findings suggest that mobilisation depends not only on individual conviction but on belonging to a group that makes public action feel expected, supported, and efficacious.

Systematic review evidence indicates that religion’s relationship to pro-environmental behaviour is complex and context-dependent, amplifying stewardship norms in some settings while attenuating climate concern in others (Morrison et al. 2015; Shrestha et al. 2025). Empirical work in Australia found that religiosity can predict climate attitudes and behaviour, even after controlling for denominational and ideological factors. Individuals with anthropocentric and monotheistic beliefs and who exhibit low humility towards the environment, religion, and the cosmos are less inclined to engage in eco-friendly actions than those who hold eco-centric, pantheistic, or animistic views (Morrison et al. 2015). These latter perspectives often embody humility regarding the human role in the world (Taylor et al. 2020).

Churchgoers often express strong environmental values, but the extent to which those values translate into action varies markedly across contexts. In Australia, NCLS Research (2021) found that while most churchgoers believe they have a responsibility to care for the environment, actual engagement in sustainability practices remains moderate, with only about 22% reporting very active involvement. By contrast, survey data from the Pew Research Center (2022a) indicate that Evangelical Christians in the USA are among the least likely to believe that climate change is human-induced, in part because environmental conditions are framed as under divine control rather than human influence. In such settings, climate action is not resisted despite concern; concern itself is attenuated. This theological framing is then reinforced by silence: Pew Research Center (2022b) reports that only 8% of US churchgoers frequently hear about climate change in sermons, while nearly half hear nothing at all. Faith-based communities therefore occupy a distinctive position in sustainability transitions: they frequently invoke stewardship as a moral duty, yet divergent theological framings and selective silence may either legitimise or inhibit pro-environmental practice (Morrison et al. 2015; Shrestha et al. 2025).

A unifying theme across these studies is that environmental action becomes more likely when responsibility is both internalised and supported—not only through policy and infrastructure, but also through cultural, moral, and relational reinforcement. The value–action and belief–behaviour gaps are therefore not simple inconsistencies; they reflect tensions between personal conviction, social identity, and systems of accountability. Bridging these gaps requires more than changing minds. It requires reshaping how people understand their role in relation to others and to the natural world, and embedding that understanding in communities that make action feel expected, legitimate, and supported. This implies that effective sustainability strategies must integrate structural reform with deep normative work—including theological and moral framing—at the congregational/institutional level. The remainder of the paper examines this dynamic empirically in the Australian Christian context.

## Materials and methods

The data displayed in this section of the report were obtained as part of the 2025 Australian Community Survey (ACS), an online survey conducted in April 2025 by NCLS Research, with a sample of *n* = 1295 drawn from a large online research panel (NCLS Research 2025; Powell and Pepper 2025). As this study is based on secondary analysis of the ACS, the religion variable was constrained by the predefined survey item (“What is your religion?”). For this article, responses to the ACS religion question were organised into four broad analytic categories: Christian attending, Christian non-attending, non-Christian, and no religion. While suitable for high-level comparison, these categories necessarily compress substantial diversity and do not fully capture Indigenous, traditional, and other culturally specific belief identifications in the Australian context. The survey was distributed by the Online Research Unit (ORU). ORU meets ISO 20252 and ISO 26362 standards for both market research and panel work. Quotas were set for age, gender, and location, derived from the 2021 Census of Population and Housing, and a quota was also set for education (given the tendency for people with higher levels of education to respond to survey invitations). The dataset is weighted to reflect the demographic profile of the Australian population aged 18+ on age, gender and education.

## RESULTS

### The faith–action disconnect in Australia

**Fig. 1:**
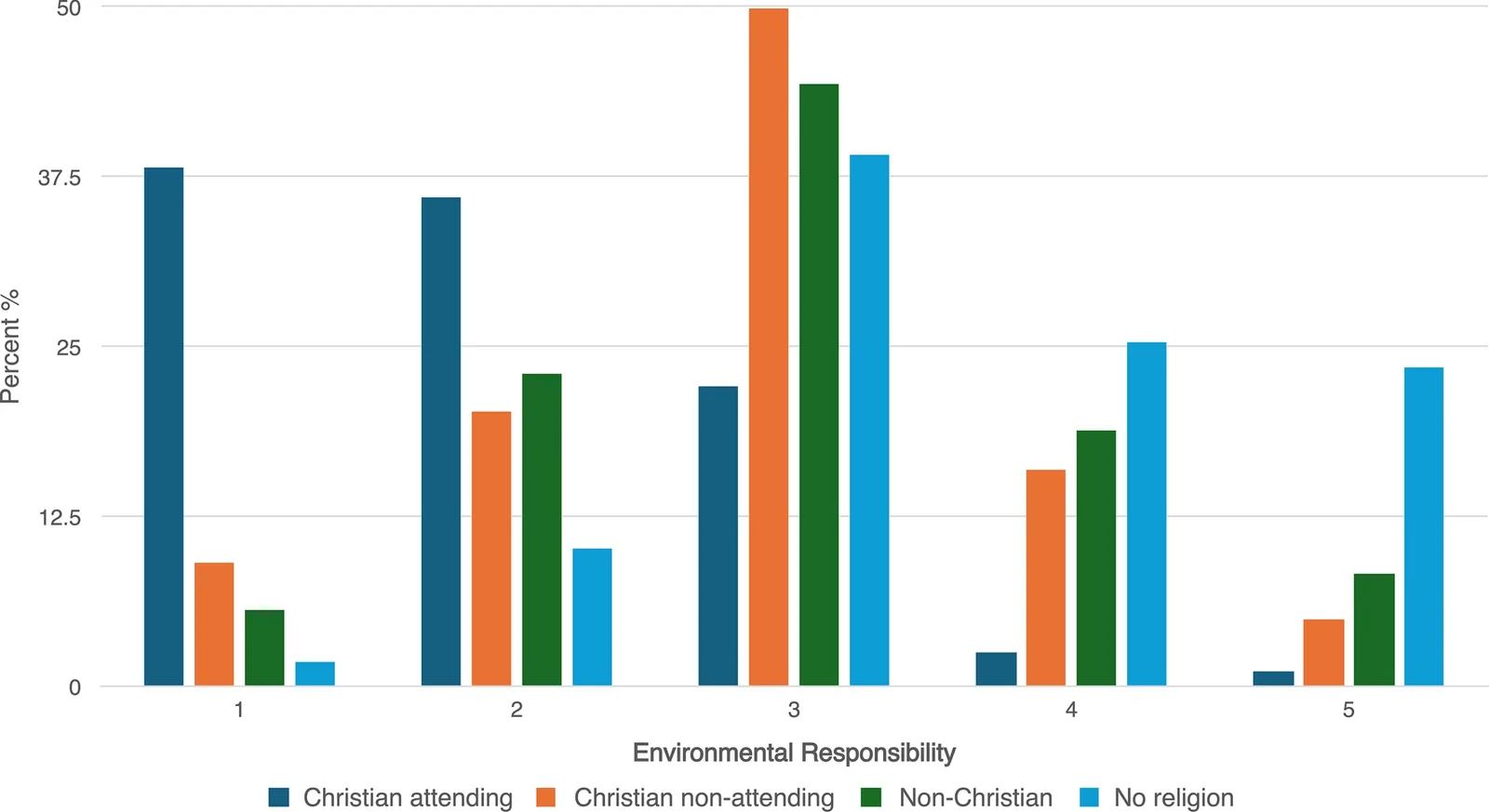
Christianity and being environmentally responsible, by religious affiliation. [Notes: Results from the 2025 Australian Community Survey (ACS) where the online research panel were asked whether they agreed or disagreed with the statement, “The Christian faith helps people to be more environmentally responsible”: 1, Strongly agree; 2, Agree; 3, Neutral/Unsure; 4, Disagree; 5, Strongly disagree. The panel were divided into four categories using the question: “What is your religion?”: Christian attending are Christians who also indicated that they attend religious services at least monthly; Christian non-attending refers to respondents who identified as Christian and indicated that they attend religious services less often than monthly; non-Christian refers to respondents who identified with a non-Christian religion; and no religion refers to respondents who reported no religion. Respondents who indicated “Prefer not to say” are not included in the results. These are broad analytic categories based on the ACS religion question and do not permit more specific identification of Indigenous or traditional spiritual belief formations. Analyses use *n* = 1268 (from a total sample of *n* = 1295).]

**Fig. 2:**
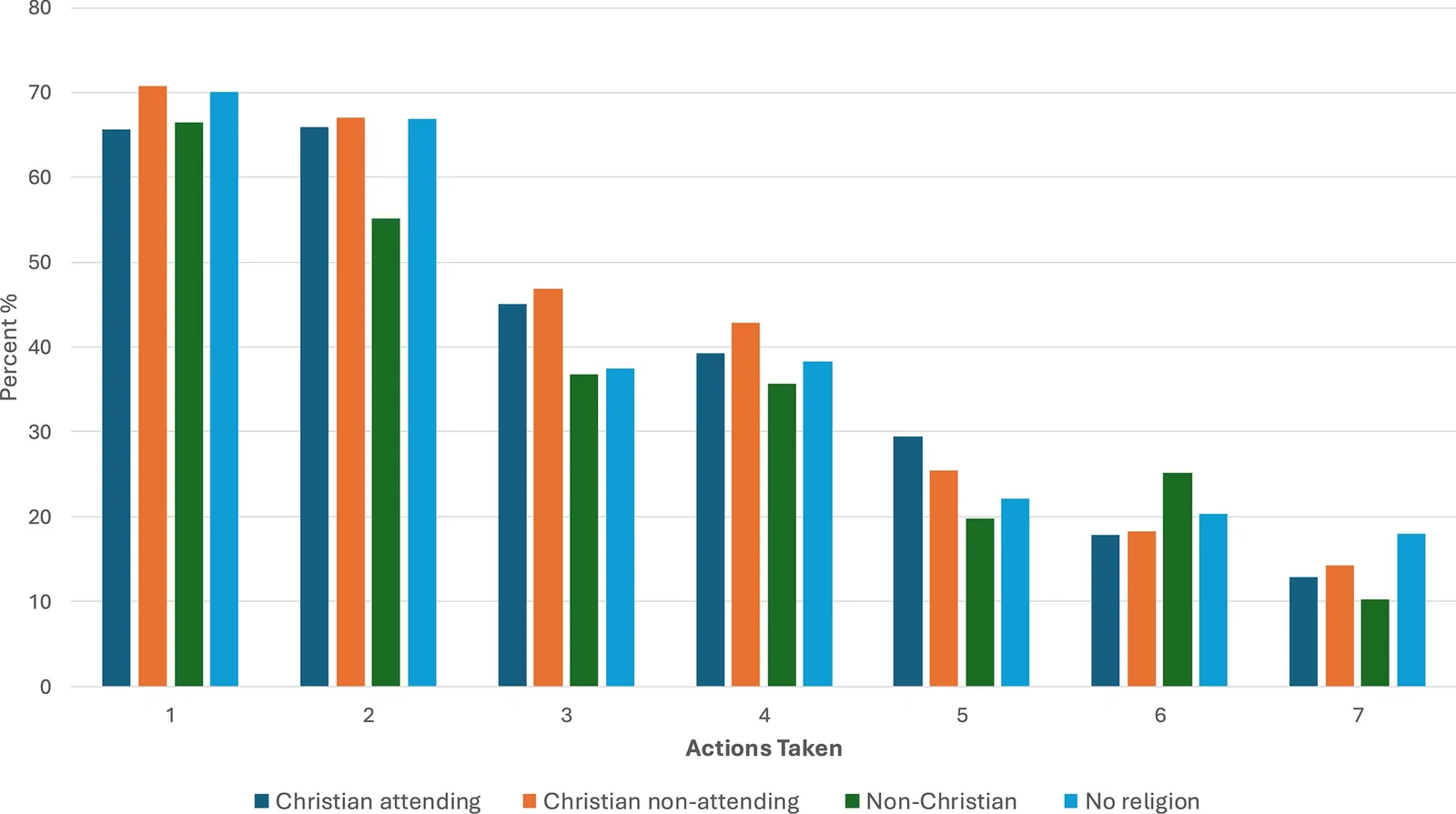
Actions taken to reduce impact on the environment, by religious affiliation. [Notes: Results from the 2025 Australian Community Survey (ACS) where the online research panel were asked, “Have you taken any of the following actions to reduce your impact on the environment?” (Please select all that apply): 1, I switch lights off at home whenever possible; 2, I recycle/compost as much household waste as possible; 3, I have reduced the amount of water I use around my home and garden; 4, I have reduced the amount of gas and/or electricity I use around my home; 5, I have installed a solar hot water system or solar panels on my home; 6, I have changed my diet (e.g. less meat, seasonal food); 7, None of the above. The panel was divided into four categories, as shown in Fig. 1.]

**Fig. 3:**
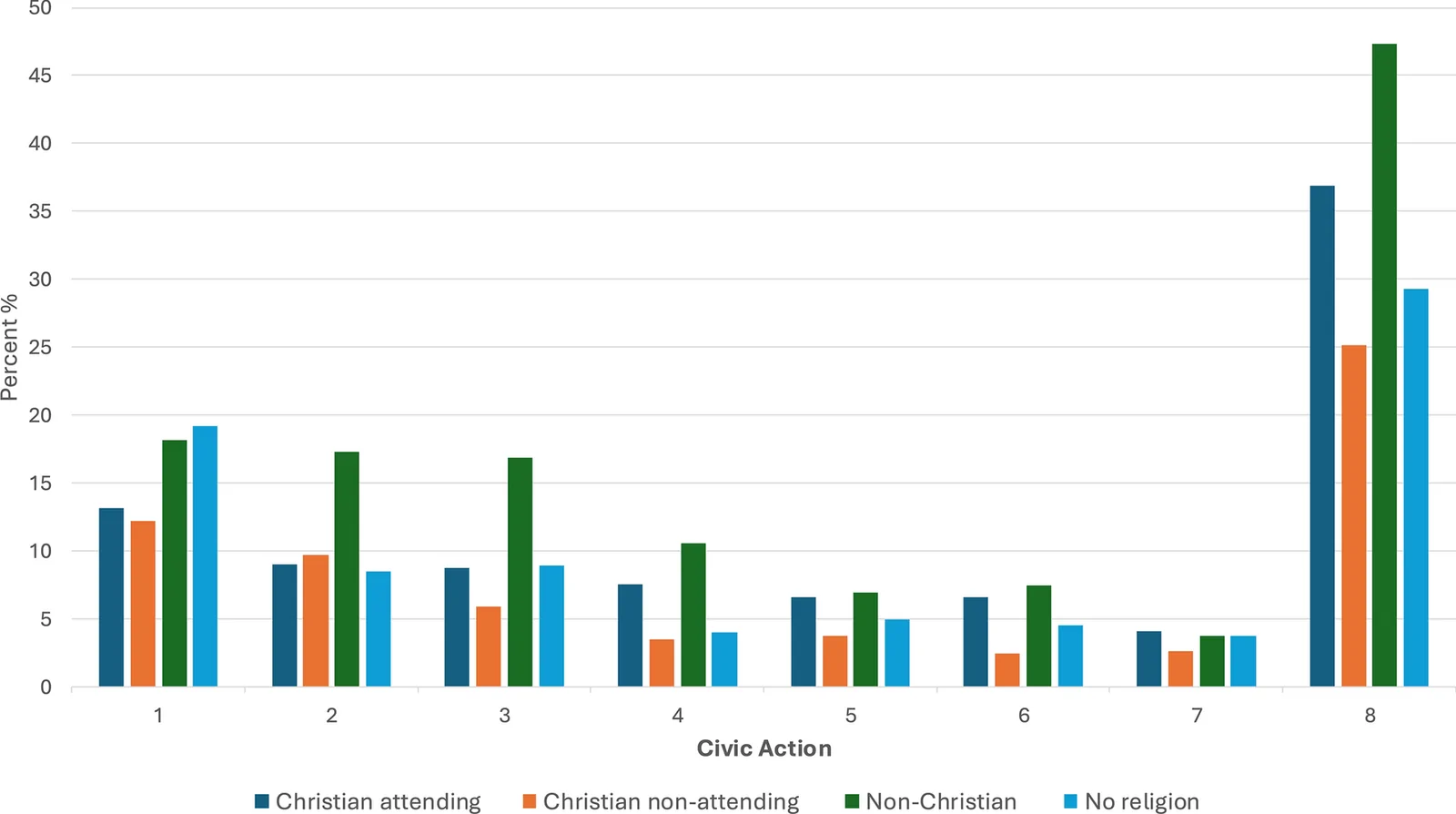
Civic actions concerning the environment taken in the last 5 years, by religion. [Notes: Results from the 2025 Australian Community Survey (ACS) where the online research panel were asked, “In the last five years, have you undertaken any of the following actions?” (Please select all that apply): 1, Voted in a government election on the basis of an environmental issue; 2, Taken part in an environmental event (e.g. Earth Hour); 3, Given money to a group that aims to protect the environment; 4, Contacted an elected government member about an environmental issue; 5, Taken part in a conservation activity (e.g. Landcare, bush regeneration); 6, Taken part in a protest or demonstration to protect the environment; 7, Been a member of an environmental group or movement; 8, At least one action (reversed from the original option of “None of the Above”). The panel were divided into four categories as in Fig. 1.]

The part of the data presented here in Figs. 1, 2 and 3 concerns the responses of the panel to questions looking at the environmental responsibility of Christians, and the practical and civic actions taken to reduce people’s impact on the environment. Figure 1 shows that Christians who are frequent church attenders (at least monthly) were twice to three times as likely as non-attenders and people of other faiths and none to agree or strongly agree that the Christian faith helps people to be more environmentally responsible (74.3%, 29.3%, 28.6% and 12% respectively). It is also notable that among those who professed no religion, 48.8% disagreed or strongly disagreed that the Christian faith was helpful in this regard. There was, however, little difference between Christians who attended religious services regularly, those who didn’t, non-Christian religions, and those who did not follow any religion, in the practical actions taken to reduce their impact on the environment (Fig. 2). Similar results for the civic actions people take in response to the environmental crisis are displayed in Fig. 3. People in non-Christian religions were the most likely to have given money to a group that aims to protect the environment (16.8%), contacted an elected government member (10.5%) and were also the group most likely to have taken at least one civic action. Those identifying as Christians were the least likely to have voted in a government election based on an environmental issue. Again, there was no evidence that Christians were taking more action on the environment than the general population.

In other data collected from the survey (see Supplementary Information 1, Tables S1–S3), the Australian population as a whole was divided on whether the Christian faith helped people be environmentally responsible (Table S1a), while age, gender, and level of education had little effect on people’s thinking (Table S1b–d). However, those who thought of themselves as religious or spiritual, who had had a mystical or supernatural experience, who prayed or meditated more, or believed in a personal God, were more convinced that Christian faith helped people to be environmentally responsible (Table S1f–i). Of course, these latter categories would overlap significantly with responses to the religion question (Fig. 1, Table S1e). In the overall data, 84.6% of Australians were found to have taken at least one practical environmental action, with switching off lights being the most common action, and changes in diet the least common (Table S2a). Older people were more likely to take environmental actions than younger people, and women were more likely than men (Table S2b and c). Level of education seemed to have little effect on actions to reduce impact on the environment (Table S2d), and similar results were found for spirituality, experience or belief in supernatural and mystical experiences, frequency of prayer and meditation, and belief about God (Table S2f–i). Again, responses to these questions would certainly overlap with those seen in Fig. 2 (tabulated in Table S2e). Only 31% of the total population of Australia had taken at least one civic environmental action, with voting based on an environmental issue being the most common (Table S3a). Younger people were more likely to have taken civic actions, but gender had little effect (Table S3b and c). Those people with a university education were more likely to have taken civic actions than those who had not (Table S3d). Spirituality, belief in mystical experiences and frequency of prayer and meditation, showed no obvious trends (Table S3f–h). Finally, it seems that those with a belief in a spirit or life force were more likely to take civic actions than those who believed in a personal God or didn’t believe in God (Table S3i). While the present data do not permit conclusions about specific Indigenous or traditional belief traditions, the pattern may be read alongside literature noting affinities between Indigenous spirituality and environmental stewardship (Luetz 2024), including within Christian ecological reflection, where *Laudato si’* recognises the environmental wisdom and relevance of Indigenous cultures (Francis 2015). These somewhat unclear patterns are also reflected in the results for religion seen in Fig. 3 (tabulated in Table S3e).

Taken together, the survey reveals a consistent pattern. Christians—especially frequent church attenders—are far more likely than other Australians to say that Christian faith helps people to be environmentally responsible. However, this confidence in faith does not translate into either higher rates of everyday pro-environmental behaviour or higher rates of civic engagement. On most practical behaviours, Christians look much like everyone else; on some civic measures, they are in fact less likely than others (including people in non-Christian traditions) to have taken action. Civic engagement overall is uncommon (only about one in three Australians has taken any civic environmental action), and is stratified more by age and education than by religious identity. Demographic factors such as age and gender explain some variation in practical behaviours, but belief in God, frequency of prayer, and reported mystical experience are not consistently associated with either practical or civic action. In short, there is no observable “Christian advantage” in environmental action: strong stated conviction among churchgoing Christians is not matched by distinctive behavioural or civic outcomes.

## DISCUSSION

### Explaining and addressing the disconnect: Pathways to civic action

Our results show a sharp asymmetry: despite markedly higher agreement that “Christian faith helps people be more environmentally responsible”, Australian Christians do not report higher rates of either everyday environmental behaviours (such as reducing waste or changing consumption) or civic environmental actions relative to others. The shortfall is most pronounced for civic behaviours that require coordination and carry public identity costs (for example, contacting elected officials or participating in conservation events or protests). In brief, beliefs are not matched by action—especially civic actions (see Sect. “Results—The faith–action disconnect in Australia” figures). We now interpret the faith–action disconnect through mutually reinforcing micro, meso, and macro hypotheses.

*Cognitive/psychological (micro):* Even when stewardship convictions are strong, two frictions seemingly depress civic mobilisation. First, individuals doubt their participation will meaningfully influence public outcomes (efficacy deficits), particularly where environmental issues feel large-scale and politically mediated (Lorenzoni et al. 2007). Second, familiar “dragons of inaction”—busyness, moral licensing (“I recycle, so I’ve done my bit”), and social risk aversion (fear of political conflict in congregations)—seem to be especially salient for civic behaviours (Gifford 2011; Kelly and Barker 2016). These frictions help to explain why household practices do not “scale up” automatically into public action.

*Cultural–theological (meso):* Congregational cultures often convey ambivalent cues about public advocacy. This may also reflect institutional caution about alienating existing or prospective members. Where climate and environmental concerns are discussed irregularly, or framed as partisan or divisive politics rather than Christian neighbour-love, injunctive norms for civic participation remain weak. Social learning theory suggests that modelling and observational learning are critical for norm internalisation; without visible exemplars, intention rarely translates into public action (Burton et al. 2015; Luetz et al. 2020). People may believe environmental responsibility is Christian, but they do not necessarily see civic engagement as “what people like us do here”.

Theological framings can also unintentionally dampen environmental engagement even where stewardship language is widely affirmed. In some Christian contexts, beliefs about divine providence or eschatological intervention may shift perceived responsibility for environmental outcomes from human agency to God’s sovereign purposes (Luetz et al. 2018; Bangert 2021). When environmental change is interpreted as primarily under divine control, the perceived urgency or efficacy of civic action may decline (Hope and Jones 2014; Hulme 2016). Interpretive tensions between “dominion” and “stewardship” readings of Genesis may further complicate the moral signal. While stewardship emphasises care for creation, dominion framings may reinforce anthropocentric priorities that weaken pro-environmental motivation in practice (Ayre 2014; Luetz and Leo 2021; Michaels et al. 2021). These dynamics may be amplified by social identity processes within some Christian communities. Where religious affiliation overlaps with political identities, environmental policy debates may be perceived through partisan frames, discouraging participation even among those who privately affirm ecological responsibility (Schwadel and Johnson 2017). The resulting pattern is not necessarily theological opposition to environmental care, but rather a cultural–theological environment in which environmental responsibility is affirmed in principle while practical and civic engagement remain limited. In such contexts, stewardship beliefs may be sincerely held without being translated into sustained environmental action, thereby helping to explain the belief–action gap identified in our survey findings.

*Structural/institutional (macro):* Across church communities, opportunity structures for low-friction civic engagement are thin. It seems plausible that many churches lack routinised pathways (templates, partnerships, roles) that seamlessly translate conviction into coordinated public acts. In addition, people often do not act because of perceived capacity constraints—they may lack time, childcare, transport, or someone to lead or organise the activity. Simply giving people more information (e.g. about climate change or sustainability) rarely overcomes these barriers; behaviour shifts most when churches make participation easy and normative—by providing practical support and visible group backing (Kollmuss and Agyeman 2002; Boyes and Stanisstreet 2011; Luetz et al. 2020).

In combination, these mechanisms clarify why civic action is where the gap is widest. Public-facing engagement depends on all three conditions at once—people must feel effective, see that “people like us do this”, and have practical support—whereas everyday environmental behaviours can be carried out privately with minimal coordination (Kollmuss and Agyeman 2002; Lorenzoni et al. 2007).

The foregoing diagnosis points to actionable levers that churches and faith-based organisations may implement, evaluate, and track longitudinally—each aligning with established mechanisms of social learning, norm change, and choice architecture/nudging (Mertens et al. 2022). The central message is not that belief is irrelevant, but that conviction without efficacy, norms, and scaffolding is brittle. Churches are uniquely positioned to supply these meso-level conditions. When civic participation becomes visibly “what we do here”, belief is more likely to predict public action—and the observed say–do gap may narrow accordingly (Kollmuss and Agyeman 2002; Lorenzoni et al. 2007; Luetz et al. 2020). Herein, leadership plays an important role. Comparative evidence indicates that evangelical responses vary by context; in Sweden, denominational leaders endorse climate science and a stewardship-based mandate for policy—often linking climate change to poverty—suggesting faith frames can legitimate public engagement even where behaviour lags elsewhere (Edvardsson Björnberg and Karlsson 2022).

**Fig. 4:**
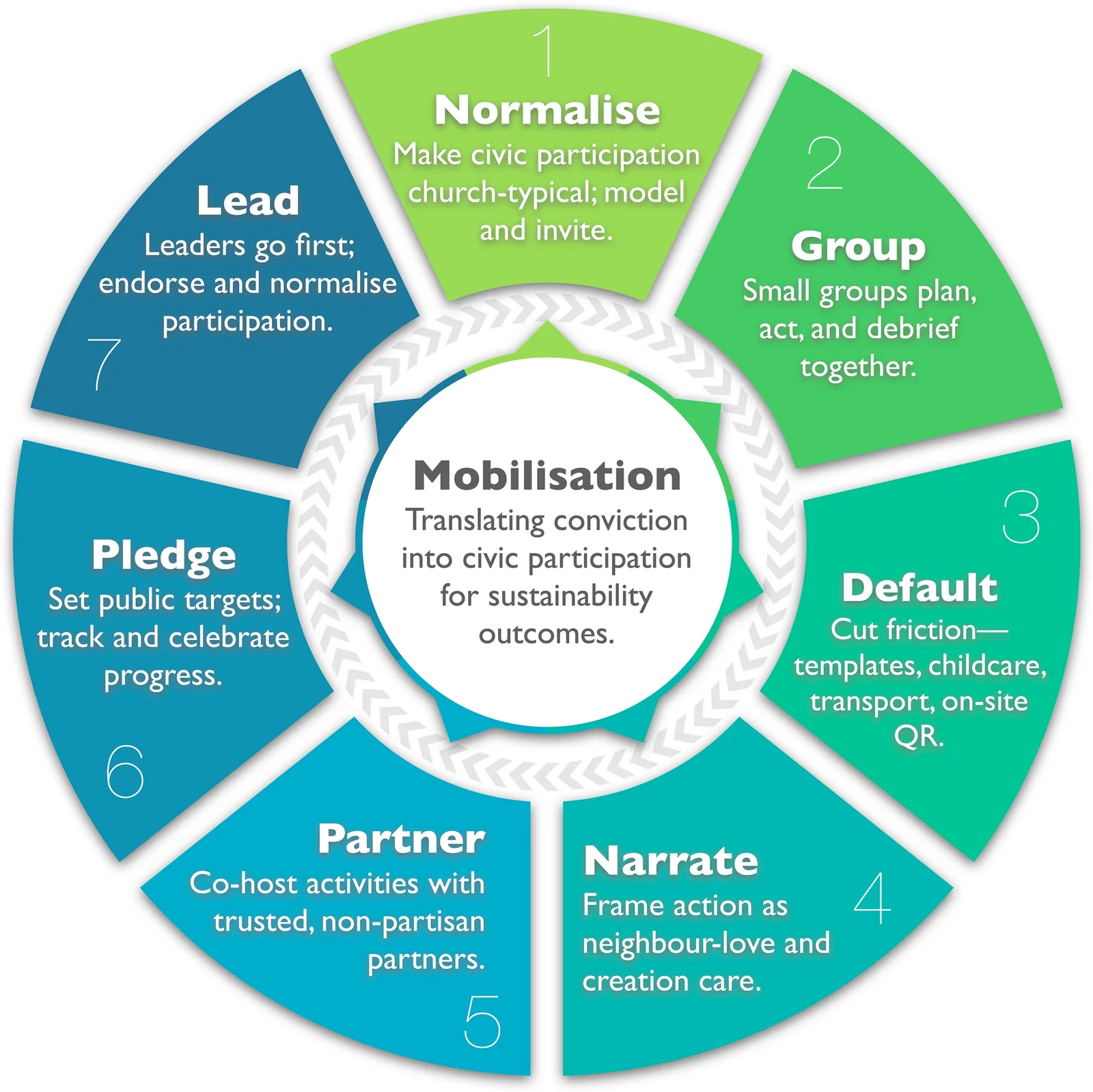
Visual synthesis of the seven congregation-level mobilisation strategies presented in Table 1, showing how faith-informed conviction may be translated into civic participation for sustainability outcomes. (*Source*: conceptual figure by authors)

Table 1 summarises seven congregation-level mobilisation strategies, each with its design principle, a concrete practice exemplar, and the behavioural rationale. Figure 4 visually synthesises these seven strategies as an integrated congregation-level mobilisation framework linking faith-informed conviction to civic participation for sustainability outcomes. The numbered segments in Fig. 4 correspond directly to strategies 1–7 in Table 1.

**Table 1:** Seven congregation-level mobilisation strategies that translate faith-informed conviction into civic participation for sustainability outcomes. Each strategy lists its design principle, a practice exemplar, the behavioural rationale, and supporting references

Mobilisation strategy	Translating conviction into civic participation for sustainability outcomes	References
(1) Make civic participation normatively ‘church-typical’	*Design principle:* Shift descriptive norms and reduce perceived social risk *Practice exemplar:* Incorporate a brief “civic moment” monthly—church leaders model a specific action (e.g., emailing a local MP), provide a QR code or printed template, and invite congregants to act during or immediately after the service *Rationale:* Visible modelling narrows norm ambiguity and lowers hassle costs at the point of decision	Burton et al. (2015), Luetz et al. (2020)
(2) Build collective efficacy in small groups	*Design principle:* Move from lone intention or individual participation to team accountability *Practice exemplar:* Home groups adopt a quarterly civic campaign (e.g., a landcare day, letter-writing round, or council submission) with defined roles and debriefs *Rationale:* Group processes and leadership cues tend to more effectively catalyse convergence in pro-environmental behaviour	Hirshleifer and Hong Teoh (2003), Burton et al. (2015), Luetz et al. (2020)
(3) Reduce hassle costs with ‘default’ participation	*Design principle:* Create choice architecture (nudging) that favours action *Practice exemplar:* Pre-printed letters and on-site e-petition kiosks; childcare during advocacy hours; transport pooling to conservation events *Rationale:* Civic behaviours are unusually sensitive to logistical frictions; defaults and convenience increase intention–behaviour conversion	Kollmuss and Agyeman (2002), Kelly and Barker (2016), Mertens et al. (2022)
(4) Anchor public action in Christian narratives	*Design principle:* Identity-consistent framing that integrates belief and practice *Practice exemplar:* Short testimonies from respected church members who frame civic action as neighbour-love and stewardship of nature; a brief sermon series linking “creation care” with local justice outcomes *Rationale:* Narrative integration strengthens injunctive norms and reduces the perceived sacred–secular divide	Bomberg and Hague (2018), Malcolm and Scott (2025)
(5) Broker partnerships and advance ready-made opportunities	*Design principle:* External organisations provide scale, continuity, and non-partisan legitimacy *Practice exemplar:* Memoranda of understanding with local conservation groups or councils to co-host recurring activities and training; an annual church–council forum to surface place-based priorities *Rationale:* Partnerships substitute for missing organisational capacity and provide a reliable pipeline of civic opportunities	White et al. (2019), Whitmarsh et al. (2021)
(6) Use public commitments and feedback loops	*Design principle:* Commitment devices plus progress salience *Practice exemplar:* Set a modest annual civic target (e.g., constituents’ contacts with MPs); publish monthly dashboards in the foyer and newsletter; celebrate milestones *Rationale:* Social influence and real-time feedback establish and consolidate emerging norms and sustain participation beyond one-off events. Public commitments (congregation-level targets) and regular progress feedback harness social influence and responsibility to convert intentions into repeated action	Gifford (2011), Luetz et al. (2020), Cooper et al. (2024)
(7) Empower leadership and liturgy as multipliers	*Design principle:* When church leaders publicly endorse and model the action, congregants perceive less risk and are more likely to participate *Practice exemplar:* If the senior pastor says, “We’re emailing our councillor today about the creek restoration—here’s the QR code; I’m sending mine now”, people see the action as approved and safe, and more of them do it on the spot *Rationale:* Leadership signals reduce perceived controversy and clarify congregational norms	Nelson and Luetz (2019), Zhang et al. (2024)

## Conclusion

### The way ahead

This paper documents a clear belief–action gap among Australian Christians, with the shortfall largest for civic engagement. By triangulating micro (efficacy and “dragons of inaction”), meso (congregational norms, leadership, and framing), and macro (institutional scaffolding) explanations, we extend sustainability scholarship beyond individual attitudes to the meso-level infrastructures that convert conviction into public action. Our contribution is twofold. Conceptually, we recast churches as mobilising institutions whose norms, narratives, and routines can either stall or catalyse civic participation. Practically, we translate this analysis into seven testable mobilisation levers that congregations and policy partners can implement and evaluate.

*Study limitations:* The study draws on cross-sectional, self-reported measures. The religion groupings available for analysis are broad and do not permit disaggregation of more nuanced spiritual, Indigenous, or traditional belief formations. Accordingly, the categories “non-Christian” and “no religion” should not be interpreted as exhaustive or culturally neutral containers for all non-Christian forms of belief, and the findings do not support specific conclusions about Indigenous Australians or distinct traditional belief communities. A further limitation concerns the design of the environmental and civic action items, some of which may privilege respondents with greater household resources, financial capacity, or control over housing, and may better capture behavioural change than long-standing low-impact lifestyles. Likewise, civic items such as donating money to environmental causes may partly reflect capacity to give and may therefore under-represent environmentally engaged respondents with lower incomes. This source of potential bias cannot be fully resolved within the present dataset and should be considered when interpreting the observed patterns. Future research could address this more directly by using measures less dependent on financial capacity and by examining socioeconomic differences in environmental engagement more explicitly. More broadly, the study cannot establish causality and may over- or under-estimate the prevalence of civic participation.

More specifically, the present survey divided respondents into four categories with regard to their religious affiliation: “Christian attending”, “Christian non-attending”, “non-Christian”, and “no religion”. The survey was conducted in the context of a much wider survey of Australian opinions on various topics, and made no attempt to subdivide these categories into denominations in the case of the Christians, or into different religions in the case of “non-Christian”. We focused our attention on the “Christian attending” category as this was the one that answered most positively to the question of whether “The Christian faith helps people to be more environmentally responsible” (Fig. 1). We have no information on the denominational affiliations of those in the “Christian attending” category, but previous work suggests that this would be an interesting avenue for further research. For example, Pew Research Center (2022a) highlighted the fact that evangelical Protestants in the USA were the most likely religious group to be sceptical about climate change, and that this appeared to be strongly linked to their political views. It is also possible to detect differences between theological traditions (“churchmanship”) within denominations. For instance, investigating Church of England ordinands in the UK, Rushton and Hodson (2012) found that those from evangelical backgrounds had less interest in environmental concerns. In Australia, and in common with many other Western countries, non-denominational Christianity is increasing in popularity (Australian Bureau of Statistics 2022). This is a very complex picture, and it is clear that a more focused study looking specifically at the environmental views of denominations, theological traditions, and those in non-denominational churches in Australia is required. Taken together, these limitations point to several opportunities for future research.

*Future directions:* The next step is empirical. Field experiments and staggered roll-out evaluations in willing congregations can test which levers actually move behaviour. These should track simple, auditable civic metrics (for example, verified contacts with elected officials, co-hosted conservation events), apply an equity lens (for example, carers and younger adults), and compare effects across denominations and places. Future research should also examine Indigenous eco-theologies and other traditional spiritual worldviews, which are not adequately captured by the broad religion categories used in the present dataset (Luetz 2024). It could also examine how eco-theological formation within specific Christian traditions and ministry contexts shapes environmental attitudes and action, including possible intergenerational effects within church communities (Potts 2023). Mixed-methods studies can then help explain which levers mobilise which groups, and why. This research agenda will clarify where and how (and how effectively) faith communities can convert stewardship conviction into sustained civic action for the common good.

*Synthesis*: Our core contribution is to show that narrowing the conviction–action gap depends less on changing individual attitudes than on how congregations make civic participation normal, easy, and visibly supported. In practical terms, these levers are directly aligned with the Sustainable Development Goals: they strengthen SDG 16 (effective, participatory institutions) and SDG 17 (partnerships), accelerate SDG 13 (climate action), and support SDG 12 (responsible consumption), while also supporting related sustainability domains including SDG 3 (health), SDG 6 (clean water and sanitation), and SDG 11 (sustainable communities). Churches (and, by extension, other faith communities), by virtue of their trust, reach, and moral framing, are well-positioned to mobilise civic engagement for sustainability in Australia and internationally, making them vital yet underutilised actors in advancing the Sustainable Development Goals, both locally and globally. Our synthesis therefore advances sustainability science beyond attitude change and offers a roadmap that can be readily implemented (e.g. monthly “civic moments”, ready-to-use templates, dashboard feedback), translating faith-informed stewardship into measurable civic participation and tangible sustainability outcomes.

## Supplementary Information

Below is the link to the electronic supplementary material.

Supplementary file1 (XLSX 19 KB)

## Data Availability

The anonymised survey data underpinning our analysis are available as Supplementary Information 1.
